# Supplementary figures and images for: Adherent and suspension baby hamster kidney cells have a different cytoskeleton and surface receptor repertoire
Source: PLoS One. 2021 Jun 4;16(6):e0246610. doi: 10.1371/journal.pone.0246610 (PMC8177424; doi:10.1371/journal.pone.0246610)

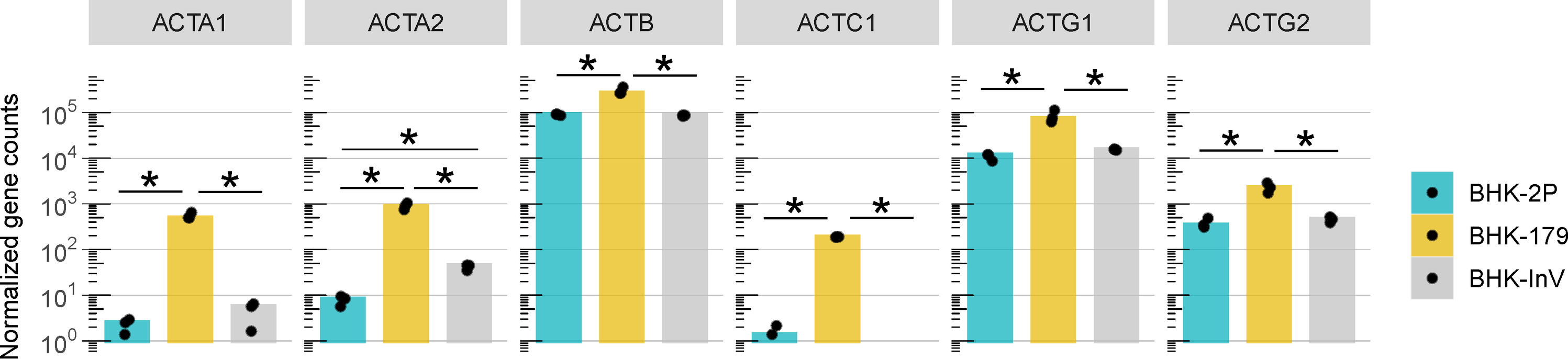

Supplement: S1 Fig — Significance code: * p < 0.05. (TIF) [file pone.0246610.s001.tif]
